# Supplementary material for: Validation of a new automated chemiluminescent anti-SARS-CoV-2 IgM and IgG antibody assay system detecting both N and S proteins in Japan
Source: PLoS One. 2021 Mar 4;16(3):e0247711. doi: 10.1371/journal.pone.0247711 (PMC7932516; doi:10.1371/journal.pone.0247711)
Supplement: Attachment — Submitted filename: Response_yokoyama_final2.pdf [file pone.0247711.s002.pdf]

Point-by-Point Responses to the Reviewers' Comments
For PONE-D-20-21741

Response to the Review Comments to the Author

Reviewer #1: The study lacks originality. Even if the new should be linked to the application to a Japonase population , this is not enough. I suggest to make it as concise report or letter and submit to a different journal.
Some techinical details should be better explained in the text such as how they tested the antibodies against the single antigen (N or S) and why they used the non reactive serum to diluite the positive serum . The kit istruction don't say this and it's a strong action since they made linearity studies.

We appreciate the critical point raised from Reviewer #1. We should admit that many articles have investigated the validity and usefulness of antibody tests, however since the clinical features of COVID-19 vary among countries, we believe it is an important task to investigate the validity and utility of antibody tests in Japan, where few researches have investigated those of antibody tests.

We apologize for the insufficient description on how to measure the antibody titers against the single antigen. We measured those antibody titers using magnetic beads coated with either antigen, respectively. The basic assay procedure is the same as the method of the original kit. We added this point to the *Material and Method* section in the revised manuscript (line 144 – 145).

We used non-reactive serum to dilute the positive serum, since a hook effect was observed in the measurement of SARS-CoV-2 IgG when we dilute the samples with saline. We added this result to the revised manuscript (line 233 – 236, Figure 2C).

Response to the Editor's comments'

In addition to the issues raised by the reviewer I would like to note the following:

1) To formally evaluate and do a verification of the analytical and clinical performance of a quantitative test specific guidelines need to be followed: e.g. CLSI EP15-A3. The authors' conclusion that the performance of the system is sufficient is not substantiated by sufficient data.

We appreciate this suggestion by the Editor. We performed the verification of precision, using two kinds of samples each assay for 5 days, 5 replicates per run, according to the CLSI document EP15-A3.

Repeatability and within-laboratory precision of all samples were lower than 5% and the upper verification limits described in the original data published by the manufacture (Ref. 20). We added these results to the revised manuscript (line 197 – 207 and Table 2).

2) How was the number of the samples chosen?

We apologize for the insufficient description on how to choose the number of the samples. We enrolled the COVID-19 subjects and the suspicious subjects, which we can collect their residual serum samples between April 22 and June 22. We added this point in the revised manuscript (line 118 – 119)

3) Basic assay background details are missing, e.g. chemiluminescence details (dye?), volumes used, calibrators, correlation between light units and AU antibody/ml.

We apologize for the insufficient description on the assay information. These assays required 5 uL of the samples and the acridinium-labeled anti-human IgM or IgG conjugate antibody was used to detect them. The unites "AU/mL" are determined by comparing the RLU detected by iFlash optical system with the cutoff calculated from the SARS-CoV-2 IgM or IgG calibrators containing anti-SARS-CoV-2 IgM or IgG chimeric antibody. We added this information to the revised manuscript (line 136 – 142).

4) Typically a new method needs to be compared to a gold standard- maybe a validated ELISA in this case? PCR is not an appropriate comparison for this assay.

We appreciate this point raised from Editor. Unfortunately, because of the biosafety facility in our laboratory, we are unable to perform a manual-based ELISA to measure the antibody titers of COVID-19 patient. Instead of ELISA, we compared the chemiluminescent anti-SARS-CoV-2 antibody tests with the Roche's COVID-19 antibody test, which received FDA emergency use authorization and was available in markets accepting the CD mark. According to the manufacturer's instruction of the Roche's test, the cutoff value for a positive COVID-19 antibody result was deemed as 1.0 cutoff index (COI). The positive and negative concordance rates between Roche's test and SARS-CoV-

2 IgM or IgG serological testing showed more than 85%, except for the positive concordance rate of SARS-CoV-2 IgM. We added these results to the *Method* section, the *Result* section, and the *Discussion* section in the revised manuscript (line 148 – 153, line 310 – 315, and line 360 – 371 and Table 4).

5) How was serum collected and processed?

We apologize for the lack of the description on how to collect serum. We collected the sera in the following steps. We received the patient's whole blood in the collection tube coated with silica and thrombin for the clinical laboratory testing. Then, the serum was separated by centrifuging at 2,300 g for 5 minutes and carried out clinical routine testing. Subsequently, we collected and stored the residual samples at -20℃ until the antibody testing. We added these pointes in the revised manuscript (line112 – 115).

6) No formal estimation of LOB and LOD, LOQ has been presented.

We appreciate this comment from the Editor. As suggested by Editor, we added the formal estimation of LoB, LoD and LoQ, according to the guidelines in CLSI document EP17. The LoD for SARS-CoV-2 IgM was 0.74 AU/mL, determined by 130 measurements with 60 blank and 70 low level replicates, and the LoB was 0.63 AU/mL. The LoD for SARS-CoV-2 IgG was 0.53 AU/mL, determined by 120 measurements with 60 blank and 60 low level replicates, and the LoB was 0.47 AU/mL. We also investigated the LoQ. We measured 7 samples of low antibody levels for SARS-CoV-2 IgM and 10 samples for SARS-CoV-2 IgG. In both assays, CVs of the samples which were lower than the LoD showed no more than 10%. Therefore, the LoQ for SARS-CoV-2 IgM was determined as 0.74 AU/mL and that for SARS-CoV-2 IgG was 0.53 AU/mL. We added those results in the revised manuscript (line 175 – 182 and line 253 – 262).

7) Linearity studies should be performed according to CLSI EP-06.

We appreciate this comment from the Editor. As suggested by Editor, we performed the linearity studies, following the guidelines in CLSI document EP06. For IgM, the method was demonstrated to be linear between 1.50 and 15.92 AU/mL since any nonlinear coefficients were not significant in this range. And for IgG, the linear range was between 2.36 and 18.30 AU/mL since any nonlinear coefficients were not

significant in this range. We added these results in the revised manuscript (line 225 – 229 and Figure 1E, F).

8) Figure 3- how were the "pre-infection" samples in the 3 patients collected?

We apologize for the insufficient information on how to collect the “pre-infection” samples. In our laboratory, the sera which completed clinical laboratory test were usually stored at -20℃ up to 3 weeks. We collected these samples since the sera which were collected and stored in our laboratory before the onset of COVID-19 symptom by chance. We added those points in the revised manuscript (line 115 – 118).

9) Line 115- typo "collected
"

We apologize for a misspelling. We have corrected this part. Thank you very much for kind suggestion (line 123).

10) A quick search shows that the YHLO chemiluminescent anti-SARS-CoV-2 Ab assay is under FDA EUA and CE marked.

We appreciate this kind information. Exactly, the YHLO chemiluminescent anti-SARS-Cov-2 Antibody assay acquired these authorizations. We believe that the evaluation in academic organizations is important to establish the clinical usefulness of these assays.

Response to the Journal Requirement

1. Please clarify whether all samples used in this study were stored biological samples, and whether samples were de-identified before researchers accessed them.

In addition, please specify whether your IRB specifically approved your informed consent plan (opt-out on website).

We have clarified these points on line 124 – 125 and line 127 – 128 in the revised manuscript.

2. Thank you for including your competing interests statement; "The present study is a collaborative research project among The University of Tokyo, Shenzhen YHLO Biotech Co., Ltd, and Medical & Biological Laboratories Co., Ltd. F. X. and F. H. are employees of Shenzhen YHLO Biotech Co., Ltd and Y. K. and J. O. are employees of Medical & Biological Laboratories Co., Ltd."

We note that one or more of the authors are employed by a commercial company: Shenzhen YHLO Biotech Co., Ltd, Medical & Biological Laboratories Co., Ltd.

Please provide an amended Funding Statement declaring this commercial affiliation, as well as a statement regarding the Role of Funders in your study. If the funding organization did not play a role in the study design, data collection and analysis, decision to publish, or preparation of the manuscript and only provided financial support in the form of authors' salaries and/or research materials, please review your statements relating to the author contributions, and ensure you have specifically and accurately indicated the role(s) that these authors had in your study. You can update author roles in the Author Contributions section of the online submission form.

Please also include the following statement within your amended Funding Statement.

"The funder provided support in the form of salaries for authors [insert relevant initials], but did not have any additional role in the study design, data collection and analysis, decision to publish, or preparation of the manuscript. The specific roles of these authors are articulated in the 'author contributions' section."

If your commercial affiliation did play a role in your study, please state and explain this role within your updated Funding Statement.

We have added the commercial affiliation in the section of funding sources (line 426 – 427) and included the requested statement (line 428 – 431).

We also revised the author contributions section (line 436 – 438) in more detail and update author roles in the online submission form.

2. Please also provide an updated Competing Interests Statement declaring this commercial affiliation along with any other relevant declarations relating to employment,

consultancy, patents, products in development, or marketed products, etc.

Within your Competing Interests Statement, please confirm that this commercial affiliation does not alter your adherence to all PLOS ONE policies on sharing data and materials by including the following statement: "This does not alter our adherence to PLOS ONE policies on sharing data and materials." (as detailed online in our guide for authors http://journals.plos.org/plosone/s/competing-interests) . If this adherence statement is not accurate and there are restrictions on sharing of data and/or materials, please state these. Please note that we cannot proceed with consideration of your article until this information has been declared.

Please include both an updated Funding Statement and Competing Interests Statement in your cover letter. We will change the online submission form on your behalf.

Please know it is PLOS ONE policy for corresponding authors to declare, on behalf of all authors, all potential competing interests for the purposes of transparency. PLOS defines a competing interest as anything that interferes with, or could reasonably be perceived as interfering with, the full and objective presentation, peer review, editorial decision-making, or publication of research or non-research articles submitted to one of the journals. Competing interests can be financial or non-financial, professional, or personal. Competing interests can arise in relationship to an organization or another person. Please follow this link to our website for more details on competing interests: http://journals.plos.org/plosone/s/competing-interests

As requested, we revised the Competing Interests Statement (line 446 – 447) and added the requested statement (line 448 – 451).

3. Thank you for updating your data availability statement. You note that your data are available within the Supporting Information files, but no such files have been included with your submission. At this time we ask that you please upload your minimal data set as a Supporting Information file, or to a public repository such as Figshare or Dryad.

Please also ensure that when you upload your file you include separate captions for your supplementary files at the end of your manuscript.

As soon as you confirm the location of the data underlying your findings, we will be able to proceed with the review of your submission.

We apologized not to include the supporting information files for the data set. We submitted the file.
