# Supplementary material for: Validation of a new automated chemiluminescent anti-SARS-CoV-2 IgM and IgG antibody assay system detecting both N and S proteins in Japan
Source: PLoS One. 2021 Mar 4;16(3):e0247711. doi: 10.1371/journal.pone.0247711 (PMC7932516; doi:10.1371/journal.pone.0247711)
Supplement: Attachment — Submitted filename: Response-yokoyamaR2.pdf [file pone.0247711.s003.pdf]

**Point-by-Point Responses to the Reviewers' Comments**
**For PONE-D-20-21741R1**

#Correction

First of all, we apologize for inappropriate interpretation in the previous version that the samples of which the titers were equal to the cutoff value had been regard as positive for SARS-CoV-2 IgM/IgG assay and Roche's kit. In this revised manuscript, we calculated these results again and as a result one sample was actually positive for Roche's kit. We revised Table 3 and corrected the numbers of samples and concordance rates in line 308-312.

*Response to the Review Comments to the Author*
*Reviewer #2: The study of Yokoyama and colleagues is about a validation of a chemiluminescent assay for detection of antibodies IgM and IgG against SARS-CoV-2. Although there is a big importance in the validation of new methods to help in Covid-19 diagnosis, the study has some deficiencies regarding the study design.*

*1) To assess the applicability of the method using real patient samples, serum samples from 26 participants with confirmed PCR and 53 from suspect cases without laboratory confirmation were included. I believe that a larger and better characterized sample panel should have been used for this evaluation. Furthermore, authors should inform readers about the demographic and clinical profile of such participants, as well as for the negative controls (collected before the period and with autoimmune diseases) in a table.*

The samples were collected from subjects with negative PCR results at the same day as the PCR test was performed and there were no subjects suspected of autoimmune diseases. For the outpatients who had visited The University of Tokyo Hospital, we have chosen the subjects without autoimmune diseases. We added this information in line 127.

*2) Still regarding the positive participants samples, analysis of the sensitivity of the assay was made according to the PCR result and the authors only give the information that serum samples were collected a few days after PCR confirmation. What is the mean number of days of collection post symptoms onset? The first reviewer also mentioned the difficulty of compare a molecular and a serological assay because of the RNA and antibodies dynamics and suggested the use of a commercial ELISA method, which was incorporated to the analysis.*

The mean days (±S.D.) between the antibody test and the onset of the symptom or the PCR test were 11.3 (±6.70) or 5.67 (±5.67) days, respectively. We added this information in line 125-127.

Of course, we admit that the difficulty in comparing a molecular and a serological assay because of the RNA and antibodies dynamics. However, the aim of the present study is not only to validate our assays but also to investigate the usefulness in the diagnosis. Therefore, we compared the results of our assay with those of PCR tests. We added the difficulty in comparing both results in the revised manuscript (line 418-421).

Regarding the comparison with an ELISA, we compared out results with the results of FDA-approved assay from Roche, since manual based ELISAs have not been established to measure the antibodies against SARS-CoV-2 in Japan in addition to the difficulty in performing manual based ELISAs from the aspects of bio safety in our laboratory.

Judging from the results in the comparison with the Roche's assay which measured antibodies without separating immunoglobulin subclasses, IgG test had a rather high concordance with the previous assay.

*3) It is not clear for me why in the comparison of CLIA and the assay from Roche, much more positive serum samples were used. Is it possible to access PCR results from these individuals? Please include more characteristics of the samples and participants.*

We apologize for the insufficient description about the characteristics of samples and participants we used in the comparison of CLIA and the assay from Roche. For this experiment (new Table 3B), we used all the serum samples which were collected from COVID-19-positive patient defined as the method section. In some cases, several serum samples collected from one patient were used.

To avoid this concern, we added the results of the concordance rates when we limited the samples only to the same samples as used in Table 2 (new Table 3A).

*4) Authors explain that samples collected before the onset of the infection were collected by chance. Did you have performed any laboratorial detection assay to guarantee that these subjects did not have current or past SARS-CoV-2 infection at that time? The same question can be applied for negative controls collected on March 2020.*

Two of those subjects had been confirmed SARS-CoV-2 PCR negative before the onset of COVID-19 symptoms. In one subject and control cases collected in March 2020, symptoms of cold were not described in medical records. In the revised manuscript, we added four cases of whom the samples collected before the onset of COVID-19 were available. One of the additional cases was confirmed PCR-negative before the onset of COVID-19 symptoms and regarding other cases, symptoms of cold were not described in medical records. We added this detail information on the subjects in line 119-122 in the revised manuscript.

*5) For the specificity analysis of the CLIA assay, is it possible to include samples from participants with confirmed coronavirus otherwise than SARS-COV-2 and also with other respiratory infections? The analysis of specificity with autoantibodies was very important.*

We agree that this is important point. At present, we did not obtain the serum of subjects infected with other coronaviruses. Instead, the manufacturer had assessed the cross-reactivity using serum samples of the subjects who had been confirmed with other respiratory infection (please see FYI Figures). These results suggested that these antibody tests did not have cross-reactivities against coronavirus other than SARS-CoV-2, such as OC43 and HKU1. These raw data, however, had not been permitted to be published by Shenzhen YHLO Biotech Co., Ltd. Therefore, we did not include these data in this paper.

*6) The method validation is well described and after first revision, it was included a validation according to a protocol (EP15-A3). Authors should rethink if it is necessary both analysis or only the validated one.*

We appreciate this kind suggestion. We will include the results of validation according to the CLSI document EP15-A3 and deleted the other results.

*7) Please include citation of Table 2A in line 196.*

We appreciate this kind suggestion. We added the citation in line 197 (new Table 1 in the revised manuscript).

*8) It is not clear for me how future researchers will interpret a negative result on CLIA*

*as a possible situation of high titers of antibodies and false negativity. How can this work on the routine clinical practice?*

We appreciate the critical point raised from the Reviewer #2. Actually, the results on Figure 2C might propose the possibility that extremely high titers of IgG might result in false-negative result. However, to our experience, IgG titers of any participants who admitted to The University of Tokyo Hospital for COVID-19 (n = 83) had never become negative for SARS-CoV-2 IgG testing during the hospitalization once the seroconversion of IgG was observed. Therefore, we think that it would not be necessary to consider false negative results of IgG due to the extremely high titers and to dilute the serum samples of negative SARS-CoV-2 IgG results on the routine clinical practice. The simultaneous measurement of IgM would help the researchers to rule out the false negativity in IgG tests, since a hook effect was not observed in the SARS-CoV-2 IgM assay. We added this point in the Discussion section (line 360-365).

*9) In figure 3, authors showed the IgM and IgG dynamics before and after PCR confirmation. I'm not sure if data of only three participants are strong for this analysis. Furthermore, how do you explain the low titers of IgM in 2 of 3 of these participants?*

In the revising the manuscript, we enrolled four more participants of whom serum samples before and after PCR conformation were available. These additional results showed that SARS-CoV-2 IgM/IgG titers were changed from the titers below the cut-off level to those over the cut-off value at all 7 participants.

Regarding the low levels of IgM titers, the titers become over cutoff value in 4 of 7 of those participants. We rearranged the scales of SARS-CoV-2 IgM and IgG titers to present the results clearly (Figure 3). We revised the manuscript (line 262-264).

*10) The concordance of CLIA assay and Roche assay was not very good mainly for IgG detection. How do you interpret this divergence? So, I don't agree with the sentence in the discussion lines 365 and 366.*

We apologize for the insufficient description about the concordance rate of CLIA assay and Roche's kit. We added the detail of the samples of which the results were not concordant (line 374-383); three samples of which the antibody titers were below 10 AU/mL by SARS-CoV-2 IgG assay and above 1 COI by Roche's kit were PCR-positive subjects, while we also observed seroconversion by SARS-CoV-2 IgG assay in the samples

collected from the three subjects after 2 days. The antibody titers of 24 samples were above 10 AU/mL by SARS-CoV-2 IgG assay and below 1 COI by Roche's kit. Among these samples, 12 cases were PCR-positive cases. In eight of these cases, the seroconversions were observed in SARS-CoV-2 IgG assay at earlier point than Roche's kit, while, in other four cases, the antibody titers measured by Roche's kit were below 1 COI although the antibody titers at the former or the latter points were above 1 COI.

Thinking these points, false-negative cases in the CLIA assay for SARS-CoV-2 IgG were fewer than Roche's assay. We added this point in the revised manuscript (line 383-384).

Also, we deleted the description in line 365 and 366 in the previous manuscript according to the suggestion from the Reviewer #2.

*11) Please include the new information of the study in the abstract section.*

We appreciate this comment from the Reviewer #2. As suggested from Reviewer #2, we added the new information in the abstract section (line 62-64).

## 6.3. Results

### Table 5-1 Assessment results of Cross Reaction

| Potential cross reaction substance | Rep. | 20200101 | | 20200102 | | 20200201 | |
|---|---|---|---|---|---|---|---|
| | | Result (AU/mL) | Judgment | Result (AU/mL) | Judgment | Result (AU/mL) | Judgment |
| Coronavirus OC43 positive serum | 1 | 1.64 | Negative | 1.42 | Negative | 1.55 | Negative |
| | 2 | 1.52 | Negative | 1.46 | Negative | 1.52 | Negative |
| | 3 | 1.44 | Negative | 1.40 | Negative | 1.52 | Negative |
| | 4 | 1.67 | Negative | 1.74 | Negative | 1.54 | Negative |
| | 5 | 1.77 | Negative | 1.40 | Negative | 1.62 | Negative |
| Coronavirus HKU1 positive serum | 1 | 1.49 | Negative | 1.77 | Negative | 1.79 | Negative |
| | 2 | 1.54 | Negative | 1.64 | Negative | 1.79 | Negative |
| | 3 | 1.65 | Negative | 1.46 | Negative | 1.59 | Negative |
| | 4 | 1.49 | Negative | 1.73 | Negative | 1.56 | Negative |
| | 5 | 1.54 | Negative | 1.62 | Negative | 1.49 | Negative |
| Influenza B virus IgM positive serum | 1 | 1.41 | Negative | 1.65 | Negative | 1.46 | Negative |
| | 2 | 1.44 | Negative | 1.59 | Negative | 1.78 | Negative |
| | 3 | 1.56 | Negative | 1.48 | Negative | 1.60 | Negative |
| | 4 | 1.59 | Negative | 1.63 | Negative | 1.61 | Negative |
| | 5 | 1.57 | Negative | 1.78 | Negative | 1.60 | Negative |
| Parainfluenza IgM positive serum | 1 | 1.69 | Negative | 1.66 | Negative | 1.53 | Negative |
| | 2 | 1.51 | Negative | 1.41 | Negative | 1.54 | Negative |
| | 3 | 1.56 | Negative | 1.63 | Negative | 1.78 | Negative |
| | 4 | 1.71 | Negative | 1.61 | Negative | 1.52 | Negative |
| | 5 | 1.68 | Negative | 1.65 | Negative | 1.57 | Negative |
| Adenovirus IgM positive serum | 1 | 1.73 | Negative | 1.79 | Negative | 1.54 | Negative |
| | 2 | 1.77 | Negative | 1.72 | Negative | 1.75 | Negative |
| | 3 | 1.56 | Negative | 1.44 | Negative | 1.60 | Negative |
| | 4 | 1.48 | Negative | 1.76 | Negative | 1.56 | Negative |
| | 5 | 1.59 | Negative | 1.44 | Negative | 1.51 | Negative |
| Varicella - zoster virus IgM | 1 | 1.68 | Negative | 1.47 | Negative | 1.70 | Negative |
| | 2 | 1.61 | Negative | 1.62 | Negative | 1.60 | Negative |
| | 3 | 1.57 | Negative | 1.44 | Negative | 1.65 | Negative |
| | 4 | 1.67 | Negative | 1.40 | Negative | 1.59 | Negative |
| | 5 | 1.75 | Negative | 1.65 | Negative | 1.78 | Negative |
| Mycoplasma pneumonia IgM | 1 | 1.62 | Negative | 1.56 | Negative | 1.70 | Negative |
| | 2 | 1.42 | Negative | 1.42 | Negative | 1.42 | Negative |
| | 3 | 1.45 | Negative | 1.44 | Negative | 1.77 | Negative |
| | 4 | 1.69 | Negative | 1.54 | Negative | 1.43 | Negative |
| | 5 | 1.49 | Negative | 1.63 | Negative | 1.66 | Negative |

| | | | | | | | |
|---|---|---|---|---|---|---|---|
| Pneumonia chlamydia IgM | 1 | 1.42 | Negative | 1.45 | Negative | 1.52 | Negative |
| | 2 | 1.68 | Negative | 1.58 | Negative | 1.52 | Negative |
| | 3 | 1.48 | Negative | 1.64 | Negative | 1.41 | Negative |
| | 4 | 1.77 | Negative | 1.60 | Negative | 1.55 | Negative |
| | 5 | 1.75 | Negative | 1.58 | Negative | 1.61 | Negative |
| EB virus capsid antigen IgM | 1 | 1.58 | Negative | 1.78 | Negative | 1.64 | Negative |
| | 2 | 1.48 | Negative | 1.73 | Negative | 1.51 | Negative |
| | 3 | 1.55 | Negative | 1.69 | Negative | 1.79 | Negative |
| | 4 | 1.55 | Negative | 1.49 | Negative | 1.43 | Negative |
| | 5 | 1.61 | Negative | 1.76 | Negative | 1.71 | Negative |
| EB virus core antigen IgM | 1 | 1.69 | Negative | 1.48 | Negative | 1.49 | Negative |
| | 2 | 1.51 | Negative | 1.66 | Negative | 1.72 | Negative |
| | 3 | 1.44 | Negative | 1.75 | Negative | 1.65 | Negative |
| | 4 | 1.79 | Negative | 1.52 | Negative | 1.77 | Negative |
| | 5 | 1.76 | Negative | 1.77 | Negative | 1.42 | Negative |
| Rubella virus IgM | 1 | 1.66 | Negative | 1.61 | Negative | 1.74 | Negative |
| | 2 | 1.60 | Negative | 1.75 | Negative | 1.79 | Negative |
| | 3 | 1.49 | Negative | 1.71 | Negative | 1.46 | Negative |
| | 4 | 1.58 | Negative | 1.45 | Negative | 1.68 | Negative |
| | 5 | 1.56 | Negative | 1.56 | Negative | 1.55 | Negative |
| Measles virus IgM | 1 | 1.57 | Negative | 1.62 | Negative | 1.51 | Negative |
| | 2 | 1.49 | Negative | 1.69 | Negative | 1.74 | Negative |
| | 3 | 1.56 | Negative | 1.65 | Negative | 1.63 | Negative |
| | 4 | 1.67 | Negative | 1.61 | Negative | 1.50 | Negative |
| | 5 | 1.68 | Negative | 1.55 | Negative | 1.53 | Negative |
| CMV IgM | 1 | 1.78 | Negative | 1.54 | Negative | 1.72 | Negative |
| | 2 | 1.77 | Negative | 1.62 | Negative | 1.68 | Negative |
| | 3 | 1.49 | Negative | 1.68 | Negative | 1.58 | Negative |
| | 4 | 1.70 | Negative | 1.58 | Negative | 1.54 | Negative |
| | 5 | 1.51 | Negative | 1.76 | Negative | 1.75 | Negative |
| Mumps virus IgM | 1 | 1.77 | Negative | 1.62 | Negative | 1.51 | Negative |
| | 2 | 1.59 | Negative | 1.56 | Negative | 1.57 | Negative |
| | 3 | 1.53 | Negative | 1.70 | Negative | 1.52 | Negative |
| | 4 | 1.76 | Negative | 1.51 | Negative | 1.55 | Negative |
| | 5 | 1.49 | Negative | 1.65 | Negative | 1.64 | Negative |
| SARS -CoV-2 IgG | 1 | 1.66 | Negative | 1.62 | Negative | 1.58 | Negative |
| | 2 | 1.78 | Negative | 1.59 | Negative | 1.63 | Negative |
| | 3 | 1.58 | Negative | 1.74 | Negative | 1.40 | Negative |
| | 4 | 1.74 | Negative | 1.46 | Negative | 1.72 | Negative |
| | 5 | 1.72 | Negative | 1.61 | Negative | 1.52 | Negative |
| Influenza A virus IgM | 1 | 1.63 | Negative | 1.59 | Negative | 1.40 | Negative |
| | 2 | 1.50 | Negative | 1.56 | Negative | 1.55 | Negative |
| | 3 | 1.54 | Negative | 1.50 | Negative | 1.72 | Negative |
| | 4 | 1.46 | Negative | 1.66 | Negative | 1.79 | Negative |
| | 5 | 1.40 | Negative | 1.67 | Negative | 1.49 | Negative |

| | | | | | | | |
|---|---|---|---|---|---|---|---|
| Respiratory syncytial virus IgM positive serum | 1 | 1.49 | Negative | 1.45 | Negative | 1.66 | Negative |
| | 2 | 1.61 | Negative | 1.70 | Negative | 1.59 | Negative |
| | 3 | 1.52 | Negative | 1.55 | Negative | 1.67 | Negative |
| | 4 | 1.58 | Negative | 1.58 | Negative | 1.73 | Negative |
| | 5 | 1.65 | Negative | 1.76 | Negative | 1.43 | Negative |
| Healthy human sample | 1 | 1.94 | Negative | 0.57 | Negative | 3.26 | Negative |
| | 2 | 2.30 | Negative | 0.64 | Negative | 0.54 | Negative |
| | 3 | 3.23 | Negative | 1.79 | Negative | 2.16 | Negative |
| | 4 | 1.30 | Negative | 1.14 | Negative | 0.77 | Negative |
| | 5 | 1.71 | Negative | 0.95 | Negative | 3.05 | Negative |
| | 6 | 2.30 | Negative | 3.48 | Negative | 3.35 | Negative |
| | 7 | 1.26 | Negative | 3.11 | Negative | 0.65 | Negative |
| | 8 | 2.59 | Negative | 3.11 | Negative | 3.28 | Negative |
| | 9 | 2.36 | Negative | 0.85 | Negative | 1.34 | Negative |
| | 10 | 1.17 | Negative | 2.48 | Negative | 3.38 | Negative |
| | 11 | 0.74 | Negative | 1.45 | Negative | 0.87 | Negative |
| | 12 | 1.78 | Negative | 0.59 | Negative | 1.83 | Negative |
| | 13 | 1.70 | Negative | 1.82 | Negative | 0.61 | Negative |
| | 14 | 0.85 | Negative | 0.85 | Negative | 0.92 | Negative |
| | 15 | 1.63 | Negative | 1.74 | Negative | 1.61 | Negative |
| | 16 | 1.16 | Negative | 1.90 | Negative | 3.43 | Negative |
| | 17 | 3.02 | Negative | 2.95 | Negative | 3.37 | Negative |
| | 18 | 1.60 | Negative | 3.39 | Negative | 0.93 | Negative |
| | 19 | 1.83 | Negative | 0.82 | Negative | 3.38 | Negative |
| | 20 | 1.34 | Negative | 3.22 | Negative | 2.62 | Negative |
| Pass/Fail | | Pass | | Pass | | Pass | |

## 6.4. Conclusion:

No cross reaction was observed in 3 lots of iFlash-SARS-CoV-2 IgM reagent kit (No.20200101, No. 20200102, No. 20200201).

### 6.3. Results

**Table 5-1 Assessment results of Cross Reaction**

| Potential cross reaction substance | Rep. | 20200101 | | 20200102 | | 20200201 | |
|---|---|---|---|---|---|---|---|
| | | Result (AU/mL) | Judgment | Result (AU/mL) | Judgment | Result (AU/mL) | Judgment |
| Coronavirus OC43 IgG | 1 | 4.1 | Negative | 4.73 | Negative | 5.07 | Negative |
| | 2 | 1.17 | Negative | 1.99 | Negative | 1.97 | Negative |
| | 3 | 1.43 | Negative | 2.14 | Negative | 2.37 | Negative |
| | 4 | 3.14 | Negative | 4.30 | Negative | 3.79 | Negative |
| | 5 | 3.99 | Negative | 5.16 | Negative | 4.73 | Negative |
| Coronavirus HKU1 IgG | 1 | 3.61 | Negative | 4.41 | Negative | 3.88 | Negative |
| | 2 | 1.87 | Negative | 2.13 | Negative | 2.11 | Negative |
| | 3 | 5.35 | Negative | 5.53 | Negative | 5.45 | Negative |
| | 4 | 3.84 | Negative | 4.23 | Negative | 4.62 | Negative |
| | 5 | 3.78 | Negative | 4.12 | Negative | 4.44 | Negative |
| Varicella-zoster virus IgG | 1 | 4.27 | Negative | 4.29 | Negative | 5.39 | Negative |
| | 2 | 3.08 | Negative | 3.54 | Negative | 3.10 | Negative |
| | 3 | 1.09 | Negative | 1.57 | Negative | 1.79 | Negative |
| | 4 | 3.01 | Negative | 4.17 | Negative | 4.01 | Negative |
| | 5 | 4.7 | Negative | 5.68 | Negative | 5.17 | Negative |
| Influenza A virus IgM | 1 | 5.43 | Negative | 6.20 | Negative | 5.69 | Negative |
| | 2 | 2.61 | Negative | 3.80 | Negative | 3.23 | Negative |
| | 3 | 3.83 | Negative | 4.89 | Negative | 3.85 | Negative |
| | 4 | 4.75 | Negative | 5.41 | Negative | 5.92 | Negative |
| | 5 | 1.2 | Negative | 1.34 | Negative | 2.16 | Negative |
| influenza B virus IgG | 1 | 3.58 | Negative | 3.80 | Negative | 4.59 | Negative |
| | 2 | 2.07 | Negative | 2.68 | Negative | 3.11 | Negative |
| | 3 | 3.06 | Negative | 3.69 | Negative | 3.34 | Negative |
| | 4 | 3.94 | Negative | 4.00 | Negative | 4.27 | Negative |
| | 5 | 1.41 | Negative | 2.17 | Negative | 2.14 | Negative |
| Parainfluenza IgG | 1 | 5.58 | Negative | 6.28 | Negative | 6.15 | Negative |
| | 2 | 1.58 | Negative | 2.28 | Negative | 1.66 | Negative |
| | 3 | 1.68 | Negative | 2.26 | Negative | 1.71 | Negative |
| | 4 | 3.63 | Negative | 4.10 | Negative | 3.98 | Negative |
| | 5 | 5.71 | Negative | 6.45 | Negative | 5.99 | Negative |
| Respiratory syncytial virus IgG | 1 | 4.09 | Negative | 4.64 | Negative | 5.28 | Negative |
| | 2 | 2.24 | Negative | 2.65 | Negative | 3.08 | Negative |
| | 3 | 4.96 | Negative | 6.13 | Negative | 5.47 | Negative |
| | 4 | 2.85 | Negative | 3.61 | Negative | 3.95 | Negative |
| | 5 | 4.95 | Negative | 6.14 | Negative | 5.74 | Negative |
| Adenovirus IgG positive serum | 1 | 1.93 | Negative | 2.60 | Negative | 2.99 | Negative |
| | 2 | 4.26 | Negative | 5.35 | Negative | 4.33 | Negative |
| | 3 | 4.6 | Negative | 5.59 | Negative | 5.56 | Negative |
| | 4 | 2.84 | Negative | 3.58 | Negative | 3.86 | Negative |
| | 5 | 1.04 | Negative | 2.08 | Negative | 1.65 | Negative |
| Mycoplasma pneumoniae IgG | 1 | 5.9 | Negative | 6.62 | Negative | 6.89 | Negative |
| | 2 | 2.68 | Negative | 3.51 | Negative | 3.16 | Negative |
| | 3 | 2.47 | Negative | 3.56 | Negative | 3.05 | Negative |
| | 4 | 1.76 | Negative | 2.14 | Negative | 2.19 | Negative |
| | 5 | 3.1 | Negative | 3.20 | Negative | 3.34 | Negative |

| | | | | | | | |
|---|---|---|---|---|---|---|---|
| IgG for chlamydia pneumoniae | 1 | 2.57 | Negative | 3.32 | Negative | 2.79 | Negative |
| | 2 | 4.73 | Negative | 5.14 | Negative | 5.03 | Negative |
| | 3 | 3.38 | Negative | 4.43 | Negative | 3.52 | Negative |
| | 4 | 1.22 | Negative | 1.88 | Negative | 1.22 | Negative |
| | 5 | 1.76 | Negative | 2.64 | Negative | 1.96 | Negative |
| CMV IgG | 1 | 5.21 | Negative | 6.03 | Negative | 5.31 | Negative |
| | 2 | 1.7 | Negative | 1.90 | Negative | 2.65 | Negative |
| | 3 | 4.47 | Negative | 4.80 | Negative | 5.57 | Negative |
| | 4 | 4.41 | Negative | 5.27 | Negative | 4.44 | Negative |
| | 5 | 5.33 | Negative | 6.27 | Negative | 5.48 | Negative |
| EB virus capsid antigen IgG | 1 | 5.82 | Negative | 5.92 | Negative | 6.74 | Negative |
| | 2 | 4.49 | Negative | 4.89 | Negative | 4.85 | Negative |
| | 3 | 2.41 | Negative | 3.15 | Negative | 2.94 | Negative |
| | 4 | 1.69 | Negative | 2.17 | Negative | 2.04 | Negative |
| | 5 | 1.42 | Negative | 1.84 | Negative | 1.61 | Negative |
| EBviruscore antigen IgG | 1 | 5.54 | Negative | 6.67 | Negative | 6.64 | Negative |
| | 2 | 1.09 | Negative | 1.65 | Negative | 1.18 | Negative |
| | 3 | 3.06 | Negative | 3.37 | Negative | 3.38 | Negative |
| | 4 | 2.32 | Negative | 3.44 | Negative | 3.40 | Negative |
| | 5 | 4.12 | Negative | 4.28 | Negative | 4.55 | Negative |
| RV IgG | 1 | 5.71 | Negative | 5.89 | Negative | 5.94 | Negative |
| | 2 | 5.96 | Negative | 6.88 | Negative | 6.50 | Negative |
| | 3 | 4.27 | Negative | 4.60 | Negative | 4.53 | Negative |
| | 4 | 5.05 | Negative | 5.60 | Negative | 5.55 | Negative |
| | 5 | 3.11 | Negative | 3.66 | Negative | 4.01 | Negative |
| SARS-CoV- 2 IgM | 1 | 3.35 | Negative | 3.84 | Negative | 3.72 | Negative |
| | 2 | 5.28 | Negative | 5.49 | Negative | 5.95 | Negative |
| | 3 | 5.66 | Negative | 6.39 | Negative | 5.69 | Negative |
| | 4 | 4.64 | Negative | 4.85 | Negative | 4.73 | Negative |
| | 5 | 4.95 | Negative | 5.58 | Negative | 5.70 | Negative |
| Normal human serum | 1 | 3.89 | Negative | 4.07 | Negative | 3.82 | Negative |
| | 2 | 1.44 | Negative | 1.38 | Negative | 1.5 | Negative |
| | 3 | 1.7 | Negative | 1.64 | Negative | 1.69 | Negative |
| | 4 | 3.42 | Negative | 3.54 | Negative | 3.49 | Negative |
| | 5 | 1.17 | Negative | 1.19 | Negative | 1.2 | Negative |
| | 6 | 4.12 | Negative | 4.3 | Negative | 3.93 | Negative |
| | 7 | 2.94 | Negative | 3.06 | Negative | 2.98 | Negative |
| | 8 | 2.74 | Negative | 2.84 | Negative | 2.63 | Negative |
| | 9 | 2.89 | Negative | 2.92 | Negative | 2.79 | Negative |
| | 10 | 1.29 | Negative | 1.31 | Negative | 1.23 | Negative |
| | 11 | 1.12 | Negative | 1.17 | Negative | 1.13 | Negative |
| | 12 | 1.89 | Negative | 1.96 | Negative | 1.9 | Negative |
| | 13 | 2.32 | Negative | 2.25 | Negative | 2.33 | Negative |
| | 14 | 4.53 | Negative | 4.32 | Negative | 4.66 | Negative |
| | 15 | 3.85 | Negative | 3.77 | Negative | 3.92 | Negative |
| | 16 | 4.76 | Negative | 4.8 | Negative | 4.57 | Negative |
| | 17 | 2.43 | Negative | 2.37 | Negative | 2.42 | Negative |
| | 18 | 2.75 | Negative | 2.78 | Negative | 2.82 | Negative |
| | 19 | 5 | Negative | 4.89 | Negative | 5 | Negative |
| | 20 | 1.84 | Negative | 1.88 | Negative | 1.86 | Negative |

**6.4. Conclusion:**

No cross reaction was observed in 3 lots of iFlash-SARS-CoV-2 IgG reagent kit (No.20200101, No. 20200102, No. 20200201).
